# Correlation between single nucleotide polymorphisms in CYP4F2 and warfarin dosing in chinese valve replacement patients

**DOI:** 10.1186/1749-8090-7-97

**Published:** 2012-09-27

**Authors:** Jie-Hui Li, Guo-Guo Ma, Shu-Qiang Zhu, Hao Yan, Yong-Bing Wu, Jian-Jun Xu

**Affiliations:** 1 Peking Union Medical College, #1, Minde Road, Donghu District, Nanchang City, Jiangxi Province, 330008, China; 2 Chinese Academy of Medical Sciences, #1, Minde Road, Donghu District, Nanchang City, Jiangxi Province, 330008, China; 3 Beijing Fuwai Hospital, #1, Minde Road, Donghu District, Nanchang City, Jiangxi Province, 330008, China; 4 Department of Internal Medicine, The Second Affiliated Hospital of Henan University of Technology, #1, Minde Road, Donghu District, Nanchang City, Jiangxi Province, 330008, China; 5 Department of Cardiovascular Surgery, The Second Affiliated Hospital of Nanchang University, #1, Minde Road, Donghu District, Nanchang City, Jiangxi Province, 330008, China

**Keywords:** Warfarin, CYP4F2, SNP, Valve, Implant, INR

## Abstract

**Background:**

Individuals with implanted mechanical valve prostheses require lifelong anticoagulation therapy with warfarin. The narrow therapeutic index of warfarin makes it difficult to dose and maintain proper anticoagulation. A number of single nucleotide polymorphisms (SNPs) affecting vitamin K or warfarin metabolism have been shown to affect warfarin dosing. Our aim was to study the effect of the CYP4F2 rs2108622-1347 (C > T) variant on warfarin dosing in Chinese patients.

**Methods:**

We studied 352 patients after heart valve replacement surgery. Warfarin dosing for patients was adjusted to achieve 1.8 ≤ INR ≤ 2.5. We determined the presence of SNPs in CYP4F2 in these patients and investigated their association with warfarin dosing.

**Results:**

We found the frequency of the CYP4F2 rs2108622 C allele was 79.5% and T-allele frequency was 20.5%. The warfarin dose requirement for CC individuals was significantly lower than that for CT or TT individuals (*P* < 0.05). TT-homozygous individuals required a 0.56 mg/day higher dose of warfarin than their CC counterparts.

**Conclusions:**

This study demonstrates that CYP4F2 rs2108622 significantly affects the warfarin dose requirement to achieve adequate anticoagulant activity in Chinese individuals. Genotyping of this SNP may allow clinicians to determine the initiation dose for patients following valve-replacement surgery in China.

## Background

In clinical practice, patients with implanted mechanical valve prosthesis are committed to lifelong anticoagulation therapy with warfarin [1]. Warfarin is a common anticoagulant with a small therapeutic safety window and there are differences in the dose requirements for different individuals. Determining the appropriate dose of warfarin for each individual—i.e., a dose sufficient to achieve the desired anti-clotting effect without producing adverse drug complications—poses as an important clinical problem for physicians and surgeons [2,3]. Currently, the available data suggest that decisions about initial warfarin dosing in different individuals are based on various factors, including age, body size, the presence of comorbidities, concomitant drug interactions, gender, diet, race, weight, and heredity [4,5].

Recent studies have analyzed genetic variations in patients taking warfarin and identified several single nucleotide polymorphisms (SNPs) that affect warfarin dosing. Two SNPs have been shown by many investigators to affect warfarin dosage, one in the vitamin K 2,3-epoxide reductase complex: VKORC1-1639 G > A, and the other involving the liver cytochrome P450 2C9 isozyme: CYP2C9 rs1057910 [6-9]. More recently, a SNP in another P450 isozyme, CYP4F2 rs2108622-1347 C > T, has been shown to be responsible for differences in warfarin dosage for many individuals [10-12]. However, different investigators have arrived at different conclusions regarding the impact of this SNP [10,12-15], and it remains a hot spot in warfarin-therapy research.

The relationship between CYP4F2 and warfarin dosing has received attention. However, there are relatively few reports of the impact of CYP42F SNP on warfarin dosing in valve-replacement patients in China. To address this issue, we analyzed the relationship between CYP4F2 genotype and warfarin dosing in Chinese patients in Jiangxi Province who underwent valve replacement surgery. We determine the CYP4F2 rs1057910 genotype and determined the warfarin dose after reaching the desired INR, to provide a basis for optimizing the warfarin dose.

## Methods

### Subjects

The study design of the current research was approved by the IRB of the Second Affiliated Hospital to Nanchang University. Informed written consent was provided by all participating patients. To be included in this study, patients were required to meet the following criteria: (1) Chinese patients diagnosed with rheumatic heart disease by echocardiography in our hospital between December 1, 2008 and December 1, 2010; (2) patients with normal preoperative prothrombin time (PT) and international normalization ratio (INR) values who were implanted with mechanical valve prosthesis after heart valve replacement surgery in our hospital; (3) between the ages of 20 to 59 years, inclusive; (4) liver function test showing normal values (albumin, globulin, bilirubin, transaminase) after at least 3 months of formal anticoagulant treatment, with 1.8 ≤ INR ≤ 2.5; (5) preoperative cardiac functions of grade I to III (NYHA classification standards); (6) postoperative echocardiography showing normal function of the mechanical valve prosthesis without atrial thrombus; and (7) patients receiving 2.5-mg warfarin tablets produced by Shanghai Pharmacy Corporation Xinyi Pharmacy Factory. Because of the potential impact of dietary sources of vitamin K, participants were instructed to avoid the consumption of animal organs, especially pig liver, during the course of the study. The participants were from the same region and shared similar dietary preferences, and no specific instructions were offered regarding other dietary sources of vitamin K, such as leafy green vegetables.

To exclude the influence of co-morbidities and concomitant drug interactions, patients meeting the following criteria were excluded from the study: (1) laboratory tests showing abnormal liver or kidney function; (2) evidence or history of hematological or other systemic diseases; and (3) individuals taking any of the following drugs: amiodarone, fluconazole, diltiazem, clofibrate, aspirin, phenylbutazone, hydroxy phenylbutazone, chloral hydrate, disulfiram, ethacrynic acid, quinidine, tolbutamide, chloramphenicol, salicylates, imipramine, metronidazole, cimetidine, broad-spectrum antibiotics, phenobarbital, glutethimide, phenytoin, vitamin K, rifampicin, chlorthalidone, spironolactone, cholestyramine, or statins.

### Collection of patient information

The following information was collected for each patient: (1) general information (name, gender, age, weight, height, admission number, contact information, home address); (2) drug status (warfarin dose, length of time on warfarin, other medications taken); (3) surgical information (surgical procedure, postoperative diagnosis); (4) medical history (cardiac function, postoperative ECG and echocardiography, and other complications).

### Methods of warfarin adjustment

#### Collection of blood samples

For each patient, two tubes containing 2 ml each of venous blood were collected within 12 hours after medication. Sodium citrate was used as an anticoagulant in the tubes. One tube was used to determine the INR as described below. The other tube was centrifuged at 4000 rpm for 30 minutes at room temperature, and the blood components were further divided into two 1.5-ml Eppendorf tubes. Tube-A was used to preserve blood cells for DNA extraction, and tube-B was used to preserve plasma in the upper layer for measuring the blood drug (R, S-warfarin) concentration. These tubes were stored at −80°C.

#### Measurement of INR

Blood samples (2 ml) containing sodium citrate were sent to the Second Affiliated Hospital of Nanchang University for INR measurement using a Sysmex CA-7000 (Sysmex, Kobe, Japan) automated coagulation analyzer with Innovin (Dade Behring, Marburg, Germany). Standard substances and quality control materials were selected from each sample group and tested to ensure a standardized testing process and reliable results.

**Figure 1:**
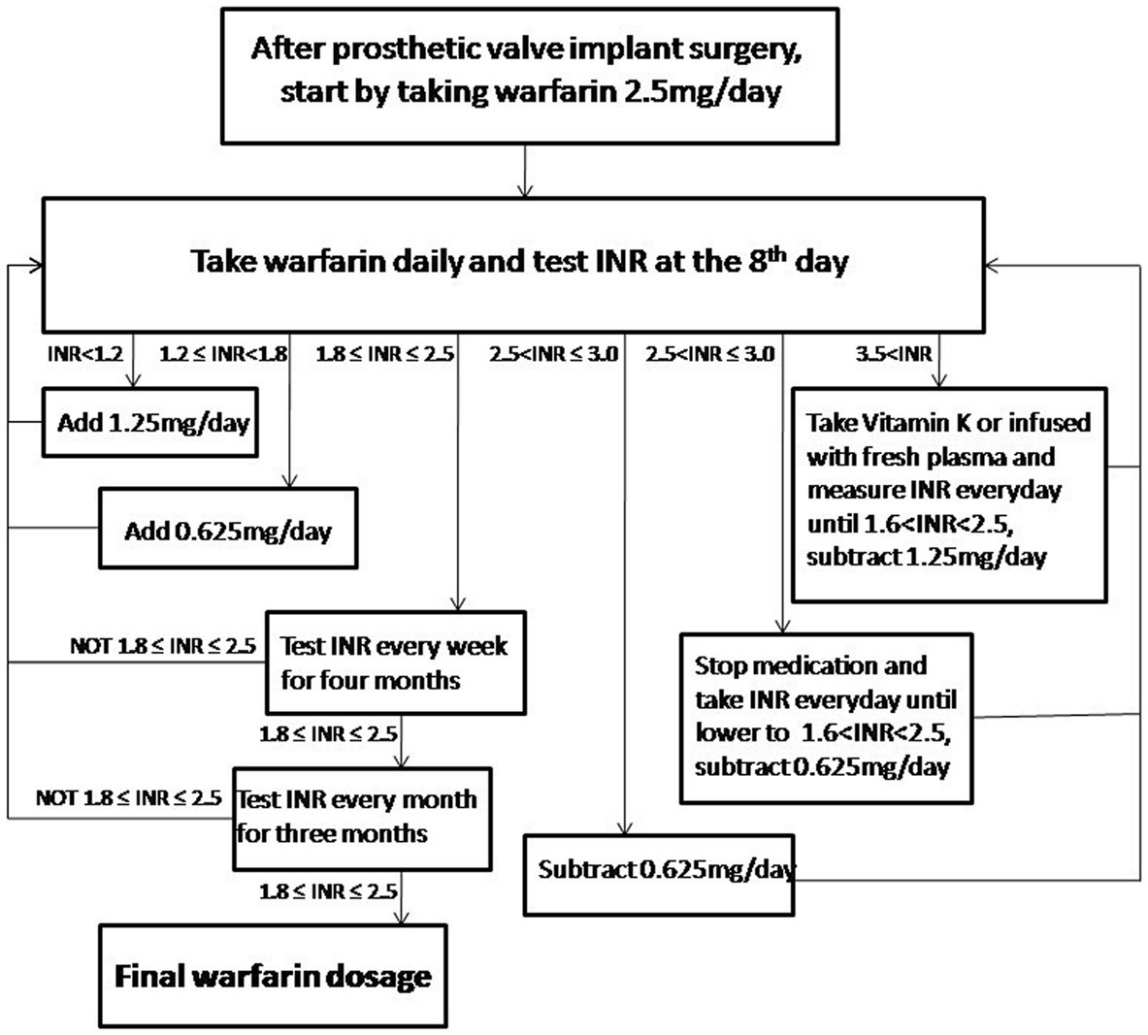
The flow chart for determing the warfarin dosage according to the Patient Medication Guide of the Cardiac Surgery Department of the Second Affiliated Hospital of Nanchang University.

Warfarin doses were adjusted according to the recommendations of the patient medication guide of the Cardiac Surgery Department of the Second Affiliated Hospital of Nanchang University, as shown in Figure 1. The timing for the adjustment of drug dosage was determined as 5 × the half-life of warfarin in blood, taken as 36 hours. Thus, dosing was adjustments were made at 8-day intervals.

### Measurement of related genes

#### DNA extraction

DNA extraction buffer of the following composition was prepared: 7.5 M guanidine hydrochloride in 0.1 M Tris–HCl, 1 M Tris–HCl, 10 mg/ml proteinase K solution, 10% (w/v) sodium dodecyl sulfate (SDS). Tube-A, containing blood cells, was thawed in a water bath at 37°C, mixed with 2 to 3 volumes of distilled deionized H_2_O (ddH_2_O). After sitting for 15 minutes, the tube was centrifuged at 8000 rpm for 5 minutes at 4°C. The supernatant was removed, and the procedure was repeated several times until the supernatant became clear. The final supernatant was removed and replaced with DNA extraction buffer. The tube was then placed in a water bath at 70°C for 30 minutes with constant gentle shaking. Following this the tube was placed in a high-speed refrigerated centrifuge and centrifuged at 10 000 rpm for 4 minutes at 4°C. The supernatant was collected and mixed with 2 volumes of dehydrated alcohol. The tube was shaken gently to promote a slow precipitation of DNA. The precipitated DNA was centrifuged in 10,000 rpm for 4 minutes to collect the DNA pellet. After washing the pellet 3 to 4 times using cold 70% ethanol, and the DNA was dried and resuspended in nuclease-free water.

#### Amplification of target gene fragments

DNA samples were amplified using polymerase chain reaction (PCR) with the following reaction system: 5 μL of 10-fold concentrated PCR buffer; 1 μL dNTPs (10 mM each); 2 μL template (genomic) DNA; 2 μL rs2108622-F (10 μM); 2 μL rs2108622-R (10 μM); 1 μL Taq DNA polymerase (2 U/μL); and 37 μL ddH_2_O. The following forward and reverse PCR primer sequences were used:

(rs2108622-F) 5′-CCCATCAACCCGTTCCCACCT-3′;

(rs2108622-R) 5′-GCCTTCTCCTGACTGCTCCCT-3′.

The PCR reaction mixture was heated to 95°C for 5 minutes and then subjected to 32 cycles of the sequence: 95°C (30 seconds), 55°C (30 seconds), 72°C (45 seconds). The product was maintained at 72°C for 7 minutes and then cooled to 4°C. Sequencing of the PCR products identified the following sequences for CYP4F2 rs2108622: CCCCGCACCTCAGGGTCCGGCCACA[C/T]AGCTGGGTTGTGATGGGTTCCGAA.

### Statistical methods

Continuous variables were presented as means and standard deviation, and were compared using ANOVA followed by Bonferroni correction. Categorical variables were summarized with frequencies and percentages, and compared using *χ*^2^ tests. Analysis of covariance was applied to test the impact of age, gender, and weight on drug dosage. Data were analyzed using SPSS 15.0 (SPSS, Inc., Chicago, IL, USA). A value of *P* < 0.05 was considered statistically significant.

## Results

INR was monitored once monthly for 3 months, in compliance with the specifications of the Chinese Society of Thoracic and Cardiovascular Surgery, the flow chart for the determination of warfarin dosage is shown in Figure 1. Patients started by taking 2.5 mg/day warfarin after the prosthetic valve implantation and the INR measured at the 8^th^ day. Patients that reached 1.8 ≤ INR ≤ 2.5 were tested for INR weekly for four months, then monthly for three months when maintained in 1.8 ≤ INR ≤ 2.5. Patients who did not reached 1.8 ≤ INR ≤ 2.5 took either decreased or increased daily warfarin dosage according to the INR measured. INR was measured again 8 days after patients took the altered warfarin dosage until INR reaching 1.8 ≤ INR ≤ 2.5.

**Figure 2:**
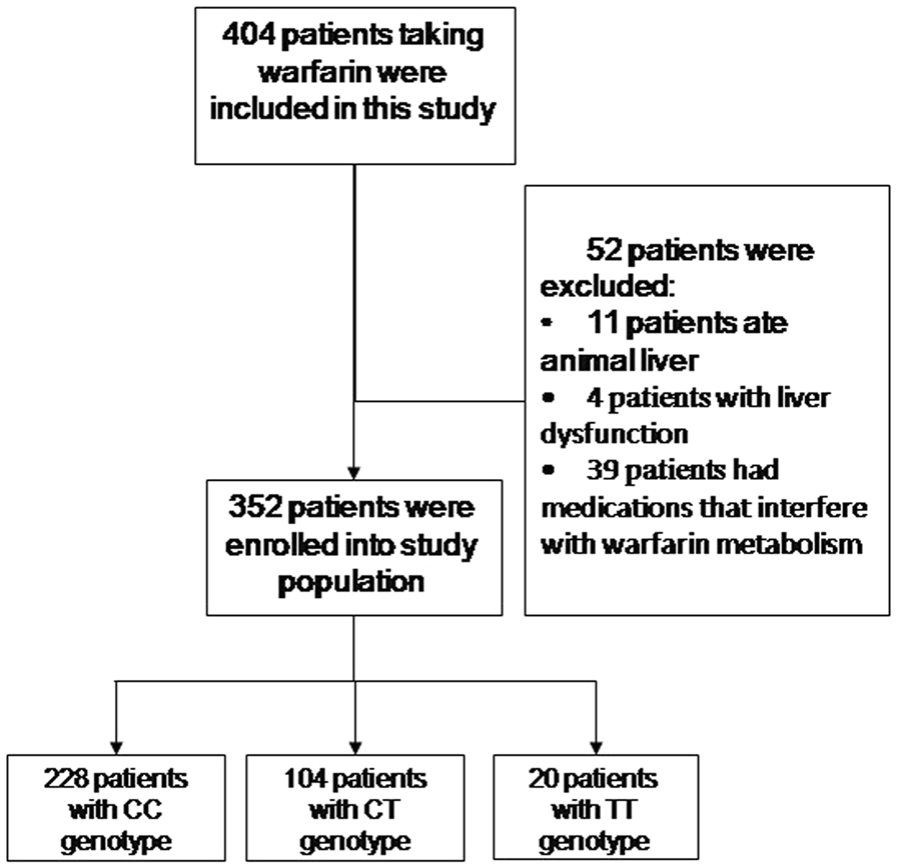
Flow chart of study enrollment and patient exclusion.

There were 404 patients recruited for this study. 52 patients were excluded for reasons listed in Figure 2. The final study included 352 patients (123 males, 229 females) who achieved 1.8 ≤ INR ≤ 2.5 and exhibited normal liver-function tests after at least 3 months of formal anticoagulant treatment. A total of 211 patients received mitral valve replacement (MVR), 69 patients received aortic valve replacement (AVR), and double valve replacement (DVR) was performed for 72 individuals. The average age and weight of patients stratified according to genotypes were: CC (41.6 ± 8.5 years, 57.4 ± 6.9 kg), CT (53.2 ± 5.2 years, 68.7 ± 6.3 kg) and TT (47.8 ± 6.5 years, 63.6 ± 5.3 kg). The mean ages and weights for each of the three groups differed significantly from one another (see Table 1), therefore correlation analyses to assess the effect of age and weight on warfarin dose were performed (see below).

**Table 1:** Patient characteristics

	**CC**	**CT**	**TT**	**P**
N	228	104	20	
Age (years)	41.6 ± 8.5	53.2 ± 5.2^*^	47.8 ± 6.5^*†^	< 0.001
Weight (kg)	57.4 ± 6.9	68.7 ± 6.3^*^	63.6 ± 5.3^*†^	< 0.001
Male	82 (36.0)	36 (34.6)	5 (25.0)	0.238
Drug dosage (mg/day)	3.1 ± 0.8	3.4 ± 0.8^*^	3.6 ± 0.9^*^	< 0.001
Blood drug conc. (ng/ml)^a^	987.1 ± 253.2	1099.4 ± 276.2^*^	1175.1 ± 314.3^*^	< 0.001

^a^ Concentration of R,S-warfarin. ^*^P < 0.05 vs CC after Bonferroni correction. ^†^*P* < 0.05 vs CT after Bonferroni correction. Allele frequency: C = 79.5%; T = 20.5%.

Among the 352 patients included in the present study, 228 patients had a CYP4F2 wild-type homozygous CC genotype, 104 patients had a heterozygous CT, and 20 patients were homozygous for the mutant TT genotype. The frequency of major C allele was 79.5%, and the frequency of minor T allele was 20.5%. The CYP4F2 2108622 obeyed the Hardy –Weinberg equilibrium (P = 0.1122).

Patients with the wild-type genotype (CC) required a daily warfarin dose of 3.1 ± 0.8 mg, but the requirement was increased significantly (P < 0.05) in heterozygous CT (3.4 ± 0.8 mg) and homozygous TT (3.6 ± 0.9 mg) individuals, corresponding to increases of 9.7% and 16%, respectively (Table 1). The changes in daily dose requirements for heterozygous and TT patients were reflected in measurements of plasma drug concentrations: (CC) 987.1 ± 253.2 ng/ml; (CT) 1099.4 ± 276.2 ng/ml, 11% increase over CC; (TT) 1175.1 ± 314.3 ng/ml, 19% increase over CC.

**Figure 3:**
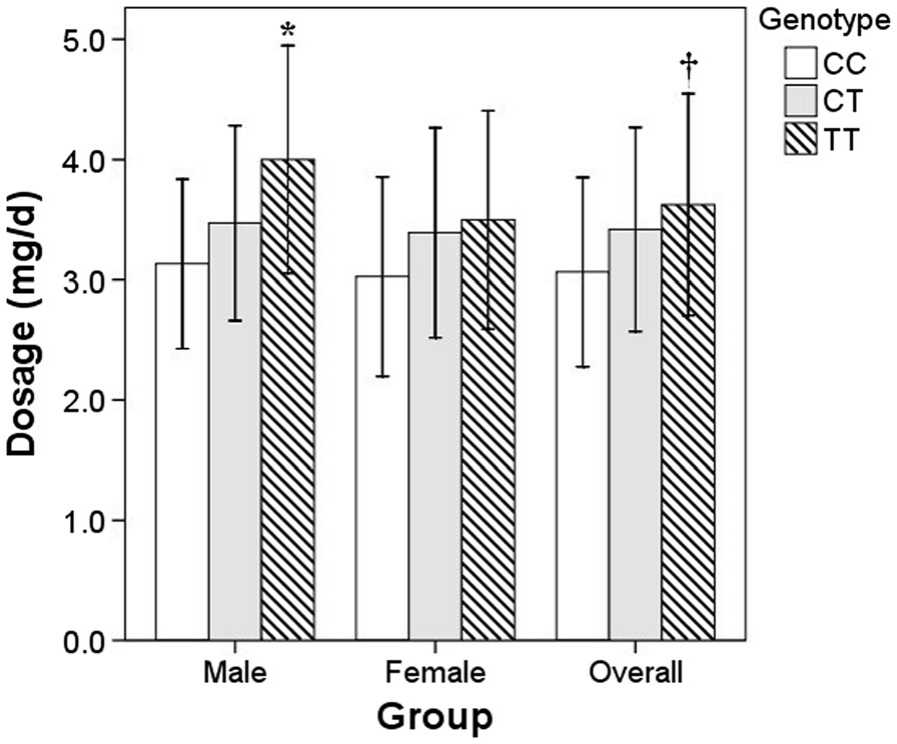
**The effect of CYP4F2 genotype to the male, female and overall patients warfarin dosage.** *P < 0.05 vs CC after adjusting for age, gender and weight and Bonferroni correction. †*P* < 0.05 vs CC after adjusting for age and weight and Bonferroni correction.

To evaluate the effect of age, weight and gender on the warfarin dosage, a correlation analysis was performed (Table 2). The analysis showed that warfarin dosage is closely associated with patient weight (r = 0.46, *P* < 0.0001), but not gender (r = 0.06, *P* = 0.2772) nor age (r = −0.04, *P* = 0.4066). Because weight is associated with the drug dosage, it was used as a covariate to evaluate the dosage distribution between genotypes (Figure 3). The weight-adjusted analysis showed that the dosage for TT patients was significantly higher than that for CC patients. The dose requirement for CT patients was moderately, but not significantly, higher than that for CC patients. When a separate analyses was performed by gender, the genotype effect was only significant in males (Figure 3).

**Table 2:** **Correlations (*P *values) between factors**

	**Age**	**Gender**	**Weight**
Dosage	−0.04 (0.4066)	0.06 (0.2772)	0.46 (<0.0001)
Age	1	−0.03 (0.6195)	0.54 (<0.0001)
Gender		1	0.16 (0.0033)

## Discussion

Among the 352 patients included in the present study, 228 patients had a CYP4F2 wild-type homozygous CC genotype, 104 patients had a heterozygous CT, and 20 patients were homozygous for the mutant TT genotype. The frequency of major C allele was 79.5%, and the frequency of minor T allele was 20.5%. This distribution is similar to that reported by others [10,15]. Our results indicate the warfarin dose requirement is increased significantly in Chinese individuals who have at least one T allele, compared to those homozygous for the C allele.

We studied patients between 20 and 59 years of age. Individuals under 20 years of age seen in our inpatient and outpatient departments rarely require valve surgery, whereas patients over 60 years of age tend to have lower rates of medical compliance, which may reflect generally lower levels of education. Poor compliance can result in serious sample loss, which can adversely affect statistical analyses. In addition, the incidence of osteoporosis in patients over 60 years of age is significantly higher than in younger patients, and undue limitation of vitamin K intake is not suitable for these patients. Finally, patient follow-ups were done through outpatient visits. Patients with grade IV cardiac function (NYHA classification) were excluded because they are difficult to follow as outpatients, and also because spironolactone, which would also interfere with our results, is often indicated for patients with grade IV heart function. Therefore, our study included only patients of NYHA grades I to III.

Recently, the Thoracic and Cardiovascular Surgery Branch of the Chinese Medical Association published “Clinical Practice Guidelines, Section of Cardiovascular Surgery”, which indicates that patients receiving biological valves after mitral valvuloplasty, mitral valve replacement, and aortic valve replacement should receive anticoagulation therapy for three months, while patients with mechanical valves should follow a course of lifetime warfarin therapy [1]. The recognized standard of anticoagulation in patients is 1.8 ≤ INR ≤ 2.5. Previous experiments have established that the different SNPs associated with CYP2C9 and VKORC1 can significantly affect the efficacy of warfarin [6-9]. This has been recognized by U.S. Food and Drug Administration (FDA), and the agency now recommends that patients adjust drug dosing according to genetic information from relevant genes [16].

In 2008, Caldwell et al. [10] reported that individuals with the CYP4F2 mutation rs2108622 (1347 C > T) required a greater average maintenance dose of warfarin. The influence of the SNP on warfarin dosing was confirmed in 2009 in a genome-wide association study in Swedish patients by Takeuchi et al. [12]. Multiple studies now indicate that the warfarin dosing requirement of individuals is increased by 4% to 13% for heterozygous CT individuals, and by 16% to 33% for homozygous for the T allele, relative to the required dose for homozygous CC individuals [10,13,17,18]. However, Kringen et al. [15] failed to detect a significant influence of CYP4F2 rs2108622 on warfarin dosing in patients from Norway. The ratios of CYP4F2 genotypes in Kringen et al. study (CC:CT:TT = 58:34.9:7.1) were similar to those in the current study (64.7:29.5:5.6). There are two possible explanations for this discrepancy. First, the Kringen study did not stratify the warfarin dosage by patient weight, which we found to be an important variable for warfarin dosing (r = 0.46, *P* < 0.0001). Second, the different result observed in the two studies may reflect differences in the genetic backgrounds of the two study populations.

There have been mixed conclusions from studies in Han Chinese patients. Lee et al. [19] detected no significant influence of CYP4F2 rs2108622 on drug dose in a general cross section of Han Chinese patients receiving warfarin treatment, but a significant effect of CYP4F2 rs2108622 was found by Cen et al. [11] in Han Chinese patients with mechanical heart valve replacement. Our data confirm those of Cen et al. [11] showing a small but significant effect of CYP4F2 rs2108622 in Han Chinese patients receiving warfarin dosing after receiving mechanical heart valves. These results support the conclusion of Liang et al. [20], who performed a meta-analysis of data from 13 different studies and found a significant effect of CYP4F2 polymorphism on warfarin dose requirements in Caucasian as well as Asian patients.

The age and weight were not comparable between patients with CC, CT and TT SNP. These difference might be due to the low patient number in TT group. We also performed a correlation analysis to assess the potential influence of age, weight, and gender on warfarin dosing. Although we did identify significant differences in age and weight for patients in the CC, CT, and TT groups, these were likely due to the relatively small study size and not to any selection bias. We found no correlation between age and warfarin dose, but did find a significant association between weight and warfarin dose. After correcting for the effect of patient weight on the warfarin dose requirement, we found a statistically significant effect of the CYP4F2 variant only in males with two T alleles. Such a gender related effect has not been reported previously. Additional studies will be required to determine if this gender-related difference is real or is an artifact of the relatively small size of this study.

The T allele of CYP4F2 rs2108622 represents a missense mutation that results in the change of valine 433 to methionine (V433M). This change in the primary structure of CYP4F2 affects enzyme activity, leading to changes in drug metabolism, physiology, and pathophysiology. Our results show that the increase doses levels of warfarin for CT and TT individuals are reflected in increased plasma levels of the drug. This indicates that the polymorphism in CYP4F2 is unrelated to a direct effect on warfarin metabolism. Mechanistic studies have revealed that CYP4F2 is involved in the oxidative degradation of vitamin K, and that the oxidative activity of the protein encoded by rs2108622 T allele is reduced compared with the product of the wild-type allele [17]. In genotyped human liver microsomal enzymes, the TT phenotype exhibited a 75% reduction in vitamin K1 oxidase activity, compared to the wild-type CC phenotype [17]. Individuals with the T allele have higher circulating levels of vitamin K, and, in turn, require higher warfarin doses to attain the anticoagulant activity achieved by lower doses in individuals with wild-type alleles. Because the influence of CYP4F2 on the warfarin dosing is smaller than that for CYP2C9 and VKORC1 [14,18,19], CYP4F2 genotyping was not included in the FDA warfarin medication guide.

The current study assessed 352 patients who were implanted with mechanical valves after heart valve replacement surgery in our hospital. These patients have to follow lifelong warfarin medication. The CYP4F2 genotype of individuals was determined and their warfarin doses were verified. INR values were determined for each patient and used to guide the patient's warfarin therapy. Because of the particularity of INR calculation method (INR = (PT_subject_/PT_normal value_)^ISI^, where ISI is the international sensitivity index), the INR value does not increase in a proportional way. Therefore grouping according to the INR value and comparison of different groups become meaningless. Because PT value is affected by various factors such as laboratory equipment, laboratory reagents, etc., there is no target value of coagulation function developed according to PT. Moreover, there is no clinical significance in the PT value after the INR meets the standard. Therefore, it was not included in the statistical analysis of the current study as an indicator.

One limitation of the present study was that we did not perform genotyping to also look for the presence of SNPs in VKORC1 and CYP2C9. Including this information would have allowed us to determine in this population the relative contribution of each of the SNPs to the overall warfarin dosing requirement. In addition, it would have been beneficial to include a cohort of patients receiving warfarin for indication other than mechanical heart valve replacement in our study.

## Conclusions

After studying 352 patients, the present study proves that genetic polymorphism in CYP4F2 significantly affects warfarin dosing in heart valve replacement patients. There are currently different views on the significance of CYP4F2 polymorphisms, and further verification of their impact on warfarin dosing is required. The pharmacogenomic study of warfarin dosing is a topic of intense research, because of the well-defined drug target and narrow therapeutic window. Though the current experiment demonstrates a correlation between CYP4F2 genetics and warfarin dosage, there are still some obstacles, such as patient privacy, that must also be overcome before the genetic information can be used to guide clinical decisions. The results of the present study should help to clarify the contribution of CYP4F2 polymorphisms on warfarin dosing and help in the formulation of future clinical guidelines for treating Chinese patients.

## Competing interests

The authors declare that they have no competing interests.

## Authors’ contributions

JL: study concepts, study design, clinical studies, data analysis, manuscript preparation, manuscript editing. GM: literature research, manuscript preparation. S-QZ: data acquisition. HY: experimental studies. Y-BW: statistical analysis. J-JX: guarantor of integrity of the entire study, study concepts, definition of intellectual content, manuscript review. All authors read and approved the final manuscript.
